# New Immunohistochemical Markers for Pleural Mesothelioma Subtyping

**DOI:** 10.3390/diagnostics13182945

**Published:** 2023-09-14

**Authors:** Iosè Di Stefano, Greta Alì, Anello Marcello Poma, Rossella Bruno, Agnese Proietti, Cristina Niccoli, Carmelina Cristina Zirafa, Franca Melfi, Maria Giovanna Mastromarino, Marco Lucchi, Gabriella Fontanini

**Affiliations:** 1 Surgical, Medical, Molecular, and Critical Care Pathology Department, University of Pisa, 56126 Pisa, Italy; iose@outlook.it (I.D.S.); marcellopoma@gmail.com (A.M.P.); gabriella.fontanini@unipi.it (G.F.); 2 Unit of Pathological Anatomy, University Hospital of Pisa, 56126 Pisa, Italy; rossella.bruno@for.unipi.it (R.B.); agneseproietti@gmail.com (A.P.); cristinaniccoli88@gmail.com (C.N.); 3 Multispecialty Centre for Surgery, Minimally Invasive and Robotic Thoracic Surgery, University Hospital of Pisa, 56100 Pisa, Italy; c.zirafa@gmail.com (C.C.Z.); franca.melfi@unipi.it (F.M.); 4 Unit of Thoracic Surgery, University Hospital of Pisa, 56126 Pisa, Italy; mgmastromarino@gmail.com (M.G.M.); marco.lucchi@unipi.it (M.L.)

**Keywords:** pleural mesothelioma, subtypes, immunohistochemistry, Mesothelin, Claudin-15, Complement Factor B (CFB), Plasminogen Activator Inhibitor 1 (PAI1), p21-activated kinase 4 (PAK4)

## Abstract

Pleural mesothelioma (PM) comprises three main subtypes: epithelioid, biphasic and sarcomatoid, which have different impacts on prognosis and treatment definition. However, PM subtyping can be complex given the inter- and intra-tumour morphological heterogeneity. We aim to use immunohistochemistry (IHC) to evaluate five markers (Mesothelin, Claudin-15, Complement Factor B, Plasminogen Activator Inhibitor 1 and p21-activated Kinase 4), whose encoding genes have been previously reported as deregulated among PM subtypes. Immunohistochemical expressions were determined in a case series of 73 PMs, and cut-offs for the epithelioid and non-epithelioid subtypes were selected. Further validation was performed on an independent cohort (30 PMs). For biphasic PM, the percentage of the epithelioid component was assessed, and IHC evaluation was also performed on the individual components separately. Mesothelin and Claudin-15 showed good sensitivity (79% and 84%) and specificity (84% and 73%) for the epithelioid subtype. CFB and PAK4 had inferior performance, with higher sensitivity (89% and 84%) but lower specificity (64% and 36%). In the biphasic group, all markers showed different expression when comparing epithelioid with sarcomatoid areas. Mesothelin, Claudin-15 and CFB can be useful in subtype discrimination. PAI1 and PAK4 can improve component distinction in biphasic PM.

## 1. Introduction

Pleural mesothelioma (PM), a rare malignant tumour of the serosae linings, involves the pleuro-pericardial surface [1]. This aetiology is mostly linked to occupational exposure to asbestos, and median survival in untreated patients is less than a year [2].

PM includes three main subtypes (epithelioid, sarcomatoid and biphasic) and can exhibit a variety of architectural patterns, often combined, including tubulo-papillary, solid and adenomatoid features and trabecular growth, as well as sarcomatoid pattern. In the same way, cellular composition can vary, showing round and monomorphic nuclei with absent atypia and inconspicuous nucleoli or displaying more aggressive features, such as rhabdoid, decidual and pleomorphic aspects. The phenotypic variety of PM underlines the necessity for a comprehensive differential diagnosis, which first includes the distinction from benign lesions and from metastatic tumours of different origin sites [3].

To date, the diagnosis of pleural mesothelioma remains difficult. Morphological evaluation may provide some essentials for a correct classification, but it must be assisted by auxiliary diagnostic techniques such as immunohistochemistry (IHC) and, more recently, molecular tests. According to the International Mesothelioma Interest Group, the PM diagnosis requires the use of two immunohistochemical markers that define mesothelial origin (such as Calretinin, Epithelial Membrane Antigen and Podoplanin) and two alternative differentiation markers (BerEp4 and monoclonal-Carcinoembryonic antigen), selecting antibodies that have sensitivity and specificity greater than 80% [3]. If there is a known history of another malignant tumour, it is useful to include the related site-specific markers [4].

The PM classification into epithelioid, sarcomatoid and biphasic depends on the morphology of the neoplastic cells, which may be epithelioid, sarcomatoid or a mixture of both. Importantly, subtype is one of the most reliable prognostic factors for PM patients, with a great impact on treatment selection [5,6,7].

Given inter- and intra-tumour morphological heterogeneity, histological subtyping can be complex. Chirieac and colleagues showed that, while the diagnosis of the sarcomatoid subtype (SS) was consistent in the comparison between diagnostic biopsies and subsequent resections, the concordance in the epithelioid subtype (ES) was lower. In fact, 20% of the epitheloid subtype was reclassified as biphasic mesotheliomas in the resection specimen, because a sarcomatoid component was evidenced [8].

The usefulness of immunohistochemical markers in the sarcomatoid setting is limited [9] and further markers are needed to identify this component (pure or mixed in the biphasic subtype—BS), owing to its prognostic and therapeutic implications.

Recent studies have focused on gene expression analysis in PM in order to highlight subtype biomarkers that can be useful to refine histological classification and patient risk stratification [10,11].

In line with these studies, our group has previously analysed the expression of 117 genes that play a key role in carcinogenesis processes. The results show that several genes were deregulated in the three PM subtypes.

In particular, five genes were differently expressed across all subtypes: *SERPINE* 1 (gene encoding for the major inhibitor of tissue plasminogen activation and urokinase) was upregulated in sarcomatoid versus biphasic and in biphasic versus epithelioid PM; *CFB* (Complement factor B), *MSLN* (Mesothelin) and *CLDN15* (Claudin-15) were all upregulated in epithelioid versus biphasic and in biphasic versus sarcomatoid PM; and *PAK4* (p21-activated kinase 4) was upregulated in biphasic PM versus the epithelioid subtype and in the latter versus the sarcomatoid PM [12].

The present study aims to validate these five IHC markers, previously selected via gene-expression analysis (Plasminogen Activator Inhibitor 1—PAI1—encoded by the *SERPINE1* gene, CFB, Mesothelin, Claudin-15, and PAK4), to improve the diagnosis and subtyping of pleural mesothelioma.

## 2. Materials and Methods

### 2.1. Study Cohorts

From the archives of our operative unit (Pathological Anatomy III, Pisa University Hospital), 103 PM samples diagnosed from 2011 to 2022 were selected. Two different cohorts were tested. The training cohort included 73 PM specimens previously investigated via gene-expression analysis [12]. In detail, 31 samples were epithelioid PMs (16 biopsies and 15 surgical resections), 25 were biphasic PMs (11 biopsies and 14 surgical resections), and 17 were sarcomatoid PMs (12 biopsies and 5 surgical resections).

The validation cohort consisted of 30 samples, distributed across the three subtypes as follows: 5 biopsies and 6 resection specimens of epithelioid PM, 9 biopsies and 2 resections of biphasic PM, 7 biopsies and 1 resection specimen of sarcomatoid PM.

All samples were formalin-fixed and paraffin-embedded (FFPE). Two pathologists (GA and ID) independently examined the specimens, confirmed the diagnosis and morphological features, and assigned the tumour grade for the ES in accordance with the fifth edition of the WHO classification of thoracic tumours [1]. The percentage of epithelioid component present in the BS samples was also determined separately by two pathologists, and the median value was considered for statistical evaluation. For immunohistochemical investigations, when possible (surgical resections), we used the most representative sample.

### 2.2. IHC Analysis and Scoring

Immunohistochemical tests were conducted on 3-μm-thick tissue sections that were prepared on slides treated with a positive charge. After deparaffinization in xylene, the sections underwent antigenic unmasking by incubation at 100 °C for 80 min in the ready-to-use solution ULTRA Cell Conditioning 1 Solution (Ventana Medical Systems). Samples were then incubated with antibodies at 36 °C for 36 min. The following antibodies were used: anti-Plasminogen Activator Inhibitor 1 (murine monoclonal antibody, clone 1D5, Abcam, 1:100 dilution); anti-Complement Factor B (rabbit polyclonal antibody, ThermoFisher, dilution 1:200); anti-Claudin15 (rabbit polyclonal antibody, Abcam, dilution 1:100); anti-p21-activated kinase 4 (Abcam, rabbit polyclonal antibody, dilution 1:600), and anti-Mesothelin (rabbit monoclonal antibody, SP74, Abcam, dilution 1:50). By the use of UltraView DAB IHC Detection Kit (Ventana Medical Systems), analysis was performed with a BenchMark ULTRA semiautomated staining instrument. After a series of washes, the samples were counterstained with Haematoxylin II and Bluing Reagent (Ventana Medical Systems), dehydrated through successive ethanol solutions of increasing concentration, and finally mounted.

Two pathologists (GA and IDS) with experience in thoracic neoplasms and blinded to all clinical and pathological data examined immunohistochemical staining independently. A third pathologist (GF) was also consulted to discuss discordant cases. In our study, Mesothelin immunohistochemical scoring was assigned considering membranous staining at any intensity in the total tumour cells, as in most of the studies in the literature [13,14]. Both the localisations, cytoplasmatic and membranous, and any intensity were considered for CFB, Claudin-15, PAI1 and PAK4 stains. In the biphasic subtype, IHC evaluation was also performed on the individual components separately.

There are no studies in the literature focused on CFB and PAK4 immunohistochemical expression in PM, and the scores and cutoffs used to assess staining for Mesothelin, Claudin-15 and PAI1 vary widely in previous reports [13,14,15,16]. Therefore, in our study, marker positivity was determined on the basis of the proportion of cells stained out of the total number of cells (Tumour Proportion Score—TPS, calculated in increments of 5%). Staining intensity (weak: 1; intermediate: 2; and strong: 3) was also determined. In order to consider the different intensities and the percentage of staining in our cohorts, we further employed a semiquantitative method, using the H-score, as previously described [13]. The H-score was determined by taking the staining intensity, most represented in the sample, and multiplying it by the percentage of positive neoplastic cells.

### 2.3. Statistical Analysis

Correlations were assessed using Pearson’s method. Two-group and three-group comparisons were performed via Mann–Whitney’s and Kruskal–Wallis’ followed by Dunn’s tests, respectively. Receiver operating characteristic (ROC) analyses were conducted in two steps following the procedures of the pROC R package v.1.18.0. Firstly, the Area Under the Curve (AUC) was computed by using the training set. The best cut-off was chosen following Youden’s method. Sensitivity, specificity, accuracy, negative predictive value (NPV) and positive predictive value (PPV) were assessed at the best cut-off value. Secondly, the cut-offs were validated on an independent cohort. Confidence intervals (CI) were assessed via 2000 bootstrap resampling. All analyses were performed in R environment (https://www.r-project.org/, v.4.2.2, last accessed on 18 April 2023).

## 3. Results

### 3.1. Patient Characteristics

The median age of the 103 PM patients was 72 years (range, 40–87). There were 83 males and 20 females with a male to female ratio of 4.15:1. For all the epithelioid PMs (42 samples), 26 (61.9%) tumours were low grade and 16 (38.1%) were high grade. The histologic features and the clinical data available are summarized in Table 1. In the biphasic subtype, the percentage of the epithelioid component was widely variable with a range of 10% to 90% and median of 40%.

### 3.2. Different Immunohistochemical Expression among Subtypes

**Figure 1:**
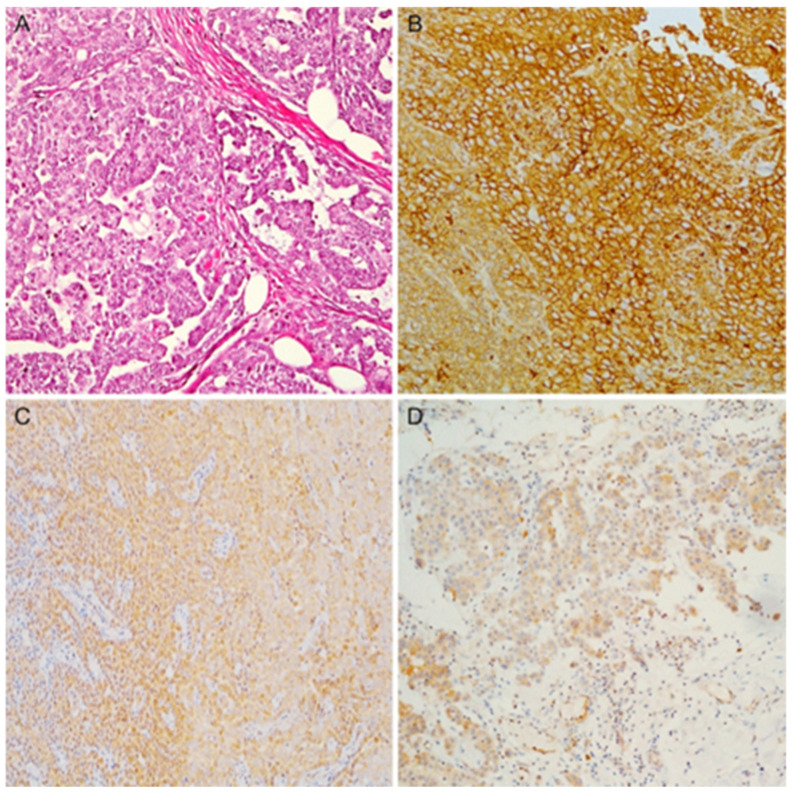
(**A**) An example of epithelioid mesothelioma (hematoxylin and eosin stain); (**B**) diffuse and strong membranous expression for Mesothelin; (**C**) Claudin-15 showing diffuse cytoplasmatic expression; (**D**) diffuse cytoplasmatic expression for CFB (magnification ×100).

Firstly, the five immunohistochemical markers were evaluated on the training cohort to assess the correlation with the gene expression data on the same samples. Mesothelin, Claudin-15 and CFB were more expressed in epithelioid PMs (Figure 1) than in sarcomatoid and biphasic PMs and more expressed in the latter than in the sarcomatoid PMs.

**Figure 2:**
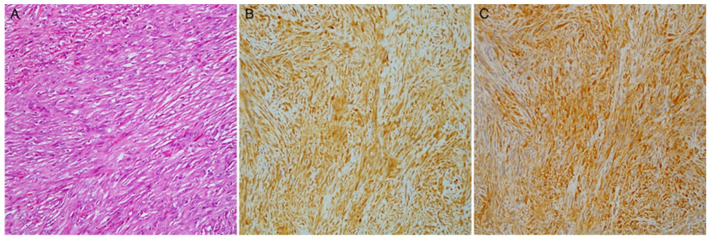
A case of sarcomatoid mesothelioma (**A**) with malignant spindle cell growth (hematoxylin and eosin stain); (**B**) PAI1 and (**C**) PAK4 diffuse expression (magnification ×100).

PAI1 staining was more evident in mesothelioma with SS (*p* < 0.0001) and BS (*p* < 0.0001) than in epithelioid MPs. No significant differences were observed in the PA1 expression between BS and SS, although assessment with TPS showed a trend for higher protein levels in SS (*p* = 0.09, Figure 2).

PAK4 showed significant results only with the H-score, according to which it was more expressed in biphasic than in epithelioid and sarcomatoid PMs, and in the SS (Figure 2) than in the ES. Subtype comparisons with both scores are reported in Table 2.

### 3.3. Score and Cut-Off Selection for Subtype Discrimination: Training Cohort

IHC expression of 4 out of 5 markers (PAI1, Mesothelin, Claudin-15 and CFB) significantly correlated with gene-expression levels. PAK4 mRNA-protein levels were not correlated. For all five markers, TPS and H-score produced similar results, although the former showed a slightly better correlation with mRNA levels. For this reason and to simplify the interpretation of IHC markers, TPS was used for all downstream analyses (Figure S1). ROC analyses were carried out for each marker in order to assess the performance in discriminating epithelioid compared to non-epithelioid PMs. Mesothelin and Claudin-15 showed the largest AUC values: 0.97 and 0.85, respectively. Lower AUC values were obtained for PAI1, CFB and PAK4, with decreasing values of 0.79, 0.76 and 0.60, respectively.

The best cut-off value was then selected for all immunohistochemical markers. Sensitivity, specificity, accuracy, PPV and NPV were computed. The cut-offs selected for Mesothelin (67.5%) and Claudin-15 (77.5%) were very satisfactory in terms of sensitivity, specificity and accuracy, showing a good ability to discriminate the subtypes.

The proposed cut-off for CFB (65%) had a good accuracy and sensitivity for ES but a lower specificity (61%). Similar results were obtained for the PAI1 cut-off (72.5%); indeed, the specificity for the non-epithelioid subtype was 0.65.

The best cut-off values (62.5%) for PAK4 showed a good sensitivity but lower accuracy and specificity (42%) for non-epithelioid PM identification. IHC expression data and cut-off performance for each marker are reported in Table S1 and Table 3.

### 3.4. Validation Cohort

We then applied the cut-offs selected in the training phase to the validation cohort. The proposed cut-offs for Mesothelin and Claudin-15 maintained a good ability to discriminate the subtypes even in the independent cohort, with accuracies of 0.83 and 0.80, respectively. For CFB, the proposed cut-off tested in the validation cohort showed an improved accuracy (0.80), with high sensitivity (0.89) and moderate specificity (0.64) in the identification of ES. Unlike the above-mentioned markers, the proposed cut-off for PAK4 (TPS ≥ 62.5%) had a lower performance in discriminating epithelioid and non-epithelioid PMs, showing an accuracy of 0.67, with a sensitivity of 0.84 and specificity of 0.36. Finally, the expression of PAI1 in the validation cohort was controversial since it was found to be more expressed in epithelioid PMs than in BS.

To evaluate the consistency of the cut-offs proposed in the training phase, the optimal cut-offs were also estimated in the validation cohort (Table 3 and Table S1).

### 3.5. Epithelioid PMs: IHC and Histological Features

Association analyses between immunohistochemical expression and tumour grade in the ES showed that PMs with higher mesothelin levels generally had a low grade (*p* = 0.02), absence of necrosis (*p* = 0.01), fewer mitoses (*p* = 0.008) and lower nuclear pleomorphism (*p* = 0.03). Epithelioid PMs with higher expression of PAK4 were frequently high-grade (*p* = 0.03). No significant results were observed between the tumour grade and immunohistochemical expression of Claudin-15 and CFB. Finally, PAI1 expression levels were higher in low-grade neoplasms (*p* = 0.04) and in cases without necrosis (*p* = 0.02).

### 3.6. Biphasic PMs: IHC and Discrimination between Components

**Figure 3:**
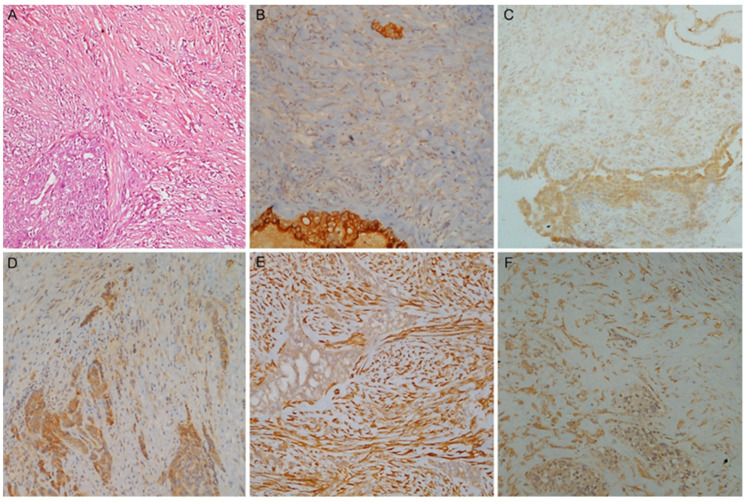
A case of biphasic mesothelioma. (**A**) Haematoxylin and eosin stain with the epithelioid (**left**) and the sarcomatoid component (**right**); (**B**) Mesothelin, (**C**) Claudin-15 and (**D**) CFB higher expression in epithelioid component; (**E**) PAI1 and (**F**) PAK4 higher expression in sarcomatoid component (magnification ×100).

To further corroborate the findings, we evaluated the IHC expression levels of the five markers in the BS to investigate differences between the two components (i.e., epithelioid and sarcomatoid) when mixed in the same tumour (Figure 3 and Figure S2). For this purpose, we also assessed the IHC expression of the markers in the individual components independently.

First, we correlated the percentage of the epithelioid component with the IHC expression of the markers in the entire tumour (i.e., epithelioid and sarcomatoid component). The staining levels of Mesothelin, CFB and Claudin-15 showed a positive correlation with the proportion of the epithelioid component. The marker assessment for each single component (i.e., either epithelioid or sarcomatoid) showed similar results. In fact, higher expression levels were observed in epithelioid compared to sarcomatoid areas.

PAI1 and PAK4 showed no significant correlation with the percentage of epithelioid component (*p* = 0.49 and *p* = 0.89, respectively). On the other hand, they showed a higher expression level in the sarcomatoid than in the epithelioid (*p* < 0.0001) component.

## 4. Discussion

The identification of IHC markers in PM has always been a topic of strong interest, especially for the differential diagnosis with benign mimickers and metastatic tumours. Nevertheless, the histological subtype represents a critical prognostic factor for PM diagnosis and influences the therapeutic approach as much as the tumour stage. According to the National Comprehensive Cancer Network guidelines, only patients with clinical stage I to IIIA, epithelioid histology and good performance status can access surgery [17].

GATA-3, MDM2, HIF1-α and CD10 have been proposed as IHC markers with potential in subtype distinction; these markers are highly expressed in non-epithelioid subtypes. Conversely, CAIX expression is virtually strong and diffuse in the ES and negative in the sarcomatoid component [18,19,20,21].

The use of these markers is limited since few studies have investigated their expression in PMs, and only a few sarcomatoid and biphasic PMs have been evaluated.

In our study, all the IHC markers tested showed a differential expression across the three subtypes, consistently with gene-expression data from the same cohort. Only the expression of PAK4 was controversial and not completely in accordance with our previous gene-expression analysis [12].

Among the IHC markers reported here, Mesothelin is one of the most studied. Consistently with other reports [14,22], it is highly expressed in ES compared to non-epithelioid subtypes. These findings hold true within BS: indeed, Mesothelin is more expressed in the epithelioid than in the sarcomatoid portion, and its expression levels positively correlate with the percentage of the epithelioid component in BS.

Higher Mesothelin levels in ES were also associated with reduced nuclear atypia, as already observed by Sandeck and colleagues [23]. In our ES series, Mesothelin expression is associated with low mitosis count, the absence of necrosis and, therefore, with low grade. These histologic features as well as increased expression of Mesothelin are associated with better prognosis in patients with epithelioid mesothelioma [1,13,22].

Interest in this marker has grown in recent years owing to anti-Mesothelin targeted therapies. Patients treated with anetumab ravtansine, an anti-Mesothelin antibody conjugated to a maytansine derivative tubulin inhibitor, experienced a better progression-free and overall survival in a preliminary evaluation. Of note, only patients with Mesothelin membrane staining observed in >50% of tumour cells were included in the study [24]. In this work, the rate of positivity for Mesothelin staining is higher than in previous studies, probably due to the use of different scores and antibodies (e.g., 5B2, MN1, MSVA-235) [14,22,25,26,27,28]. Only Vizcaya et al. used the same clone (i.e., SP74) and found similar results to ours [13].

Concerning Claudin-15, the epithelioid subtype has a higher expression than non-epithelioid PMs, in agreement with previous reports [15]. Among the non-epithelioid PMs, Claudin-15 is more expressed in BS, in contrast to Watanabe et al. However, the reason is probably due to the small sample size of the study (only eight biphasic PMs and six sarcomatoid PMs). In the biphasic group, Claudin-15 expression correlates with the percentage of the epithelioid component, and it is higher in the epithelioid compared to in the sarcomatoid fraction.

Further studies will be needed to confirm the role of Claudin-15 in PM subtyping, including its potential prognostic significance. In breast cancer, a low Claudin-15 expression is associated with triple-negative tumours, which are the most aggressive neoplastic group [29]. Indeed, some authors have suggested that decreased Claudins expression leads to cellular adhesion loss. This could represent an additional step towards epithelial-to-mesenchymal transition [30] and could explain the low Claudin-15 expression in sarcomatoid mesothelioma.

In this study, CFB expression was higher in the ES than in the non-epithelioid subtypes. CFB expression could be useful in subtype distinction, showing a different expression in the two components and a correlation with the percentage of epithelioid portion in the biphasic group.

CFB staining has not been previously evaluated in PM. In lung cancer [31] and in pancreatic adenocarcinoma [32], low CFB expression correlated with the presence of more aggressive tumours and a worse overall survival; consistently, in our cohort, SS showed the lowest CFB expression.

Previous studies have hypothesized that CFB suppression can induce cellular reprogramming (activation of a senescence-associated secretory phenotype), with the acquisition of a more aggressive morphology [33,34].

As regards PAI1, higher expression was observed in PMs with sarcomatoid features (SS and BS sarcomatoid component). However, in the validation cohort we also observed high PAI1 expression in ES. As far as we know, this is the first study to show high PAI1 expression in the PM subtype with more favourable clinical behaviour. Moreover, in ES, high levels of PAI1 are associated with features of better prognosis, i.e., low grade and absence of necrosis. On the contrary, Sidi et al. [16] showed that high PAI1 expression in PM was associated with poorer survival and lower progression-free time, but stratification by histological subtypes is absent in this study. Similarly, in other malignancies (e.g., in breast [35], oesophageal [36], gastric [37] and non-small cell lung cancer [38]), the overexpression of PAI1 is associated with poor prognosis.

Further work needs to be performed to establish PAI1′s prognostic role, despite the high PAI1 expression in PM, regardless of subtypes (63% and 66.7% in the training and validation cohorts, respectively), as this could be useful for therapeutic purposes. In this context, a preliminary in vitro study by Takayama et al. demonstrated that the use of a selective PAI1 inhibitor could suppress neoplastic progression of PM by blocking neoangiogenesis [39].

Finally, PAK4 expression does not accurately discriminate between histological subtypes; despite this, different expression levels were observed between the two components in the BS. Interestingly, epithelioid PMs with increased PAK4 expression were frequently high-grade.

To the best of our knowledge, no other studies have evaluated PAK4 expression in PM, and contrasting results have been reported in other malignancies. PAK4 overexpression is associated with poor prognosis in gastric tumours, non-small cell lung cancer, oral squamous cell carcinoma, colon cancer and clear cell renal carcinoma [40,41,42,43,44]. On the contrary, lower PAK4 expression was associated with worse prognosis in pancreatic carcinoma and endometrial cancer [22,45].

Some limitations associated with the present study should be mentioned. Firstly, with the exclusion of Claudin-15, a cut-off optimization might be necessary. Large differences in optimal cut-off were observed between training and test phases for the other markers, although the subtyping performance of mesothelin was remarkable in both phases.

Secondly, clinical information and follow-up data were not available, thus limiting the prognostic evaluation of these markers.

Despite these limitations, our study has demonstrated that Mesothelin, Claudin-15 and CFB are useful in subtype discrimination, confirming predilection for the epithelioid subtype. Given the high expression in the sarcomatoid component, PAK4 and PAI1 could support the diagnosis of non-epithelioid subtypes, since these components require new immunohistochemical markers for identification.

Further studies should evaluate the usefulness of these markers in subtyping and in the prognostic stratification of PM patients.

## 5. Conclusions

The discrimination of PM subtypes can be challenging, particularly on small biopsies. In this study, we defined five immunohistochemical markers useful to subtype epithelioid, biphasic and sarcomatoid PM, two of which (CFB and PAK4) have never been tested. In detail, Mesothelin, Claudin-15 and CFB can discriminate subtypes with good sensitivity and specificity. PAI1 and PAK4 may be useful in the diagnosis of biphasic mesothelioma since they are frequently expressed in the sarcomatoid portion. The availability of immunohistochemical markers specific for PM subtypes can greatly improve PM patients’ management, allowing better risk stratification and treatment definition.

## Figures and Tables

**Table 1:** Clinicopathological characteristics of patients with pleural mesothelioma.

**Characteristics**	**Training Cohort (*n* = 73)**	**Validation Cohort (*n* = 30)**
Age, years, median (range)	71 (40–85)	75 (54–87)
Sex, male, *n* (%)	58 (79.5)	25 (83.3)
Mesothelioma Subtype		
Epithelioid, *n* (%)	31 (42.5)	11 (36.7)
Biphasic, *n* (%)	25 (34.2)	11 (36.7)
Sarcomatoid, *n* (%)	17 (23.3)	8 (26.6)
**Epithelioid subtype (*n* = 42)**	**Training Cohort (*n* = 31)**	**Validation Cohort (*n* = 11)**
High grade, *n* (%)	12 (38.7)	3 (27.3)
Mitosis number score		
1 (≤1 mitosis/2 mm^2^)	11 (35.5)	5 (45.4)
2 (2–4 mitoses/2 mm^2^)	15 (48.4)	3 (27.3)
3 (≥5 mitoses/2 mm^2^)	5 (16.1)	3 (27.3)
Nuclear atypia score		
1	8 (25.8)	3 (27.3)
2	17 (54.8)	5 (45.4)
3	6 (19.4)	3 (27.3)
Necrosis presence	13 (41.9)	4 (36.4)

**Table 2:** Immunohistochemical expression among subtypes (training cohort = 73).

	Scores	CFB Median (IQR)	Mesothelin Median (IQR)	Claudin-15 Median (IQR)	PAI1 Median (IQR)	PAK4 Median (IQR)
ES	TPS H-score	70 (55–90) 120 (55–180)	92.5 (81.25–95) 270 (190–285)	85 (70–95) 190 (130–210)	60 (50–80) 120 (82.5–170)	70 (52.5–82.5) 120 (85–160)
BS	TPS H-score	60 (30–70) 80 (40–120)	50 (30–70) 117.5 (60–187.5)	70 (60–75) 150 (130–195)	85 (80–90) 210 (160–270)	80 (70–90) 210 (180–240)
SS	TPS H-score	10 (5–20) 10 (5–20)	0 (0–10) 0 (0–15)	35 (30–60) 60 (35–80)	90 (80–95) 210 (190–255)	70 (65–90) 160 (110–195)
ES vs. BS *p*-value	TPS H-score	0.04 0.05	<0.0001 <0.0001	0.0006 0.17	0.001 <0.0001	0.05 <0.0001
ES vs. SS *p*-value	TPS H-score	<0.0001 <0.0001	<0.0001 <0.0001	<0.0001 <0.0001	<0.0001 <0.0001	0.23 0.04
BS vs. SS *p*-value	TPS H-score	0.0003 0.0001	0.0001 <0.0001	0.004 <0.0001	0.09 0.23	0.25 0.03

CFB: Complement factor B; PAI1: Plasminogen activator inhibitor 1, PAK4: p21-activated kinase 4; ES: epithelioid subtype; BS: biphasic subtype; SS: sarcomatoid subtype; IQR: interquartile range; TPS: Tumour Proportion Score.

**Table 3:** Cut-off selection and performance among subtypes: training and validation cohorts.

**Training Cohort**					
	**Mesothelin**	**Claudin-15**	**CFB**	**PAI1**	**PAK4**
Cut-off	67.5%	77.5%	65%	72.5%	62.5%
AUC	0.97 (0.93–0.99)	0.85 (0.75–0.93)	0.76 (0.64–0.87)	0.79 (0.67–0.89)	0.60 (0.47–0.73)
Sensitivity	0.88 (0.76–0.98)	0.88 (0.57–1)	0.86 (0.55–1)	0.88 (0.62–0.98)	0.86 (0.43–0.98)
Specificity	1 (0.94–1)	0.71 (0.51–0.94)	0.61 (0.32–0.90)	0.65 (0.42–0.84)	0.42 (0.23–0.81)
Accuracy	0.93 (0.86–0.97)	0.81 (0.70–0.89)	0.75 (0.66–0.82)	0.77 (0.67–0.85)	0.67 (0.58–0.77)
NPV	0.86 (0.76–0.97)	0.83 (0.61–1)	0.76 (0.57–1)	0.79 (0.60–0.95)	0.70 (0.50–0.89)
PPV	1 (0.95–1)	0.80 (0.72–0.94)	0.76 (0.67–0.90)	0.76 (0.68–0.87)	0.67 (0.61–0.77)
**Validation Cohort**					
	**Mesothelin**	**Claudin-15**	**CFB**	**PAI1**	**PAK4**
AUC *	0.98 (0.92–1)	0.84 (0.66–0.97)	0.80 (0.59–0.97)	NA	0.75 (0.57–0.90)
Sensitivity *	0.79 (0.58–0.95)	0.84 (0.68–1)	0.89 (0.74–1)	NA	0.84 (0.68–1)
Specificity *	0.91 (0.73–1)	0.73 (0.45–1)	0.64 (0.36–0.91)	NA	0.36 (0.09–0.64)
Accuracy *	0.83 (0.70–0.93)	0.80 (0.63–0.93)	0.80 (0.67–0.93)	NA	0.67 (0.53–0.80)
NPV *	0.71 (0.56–0.92)	0.73 (0.50–1)	0.78 (0.55–1)	NA	0.57 (0.25–1)
PPV *	0.94 (0.81–1)	0.84 (0.71–1)	0.81 (0.70–0.95)	NA	0.70 (0.60–0.81)
**Best Cut-Off on Validation Cohort**					
	87.5%	75%	47.5%	65%	77.5%

AUC: Area Under the Curve; NPV: Negative Predictive Value; PPV: Positive Predictive Value; CFB: Complement factor B; PAI1: Plasminogen activator inhibitor 1, PAK4: p21-activated kinase 4. NA: Not Available because of inconsistent expression levels with the training cohort. *: Calculation based on the training cohort.

## Data Availability

The original contributions presented in the study are included in the article and supplementary files. Further inquiries can be directed to the corresponding author.

## Supplementary Materials

The following supporting information can be downloaded at: https://www.mdpi.com/article/10.3390/diagnostics13182945/s1, Figure S1: Correlation between immunohistochemical results and gene expression levels; Figure S2: Immunohistochemical expression in the two components of biphasic subtype and correlation with the percentage of epithelioid component; Table S1: High immunohistochemical expression (>cut-off) among subtypes: training and validation cohort.
