# Supplementary material for: New Immunohistochemical Markers for Pleural Mesothelioma Subtyping
Source: Diagnostics (Basel). 2023 Sep 14;13(18):2945. doi: 10.3390/diagnostics13182945 (PMC10529020; doi:10.3390/diagnostics13182945)
Supplement: Supplementary file 1 [file diagnostics-13-02945-s001.zip › Supplement Figure Legends.pdf]

Supplement Figure S1: Correlation between immunohistochemical results and gene expression levels.

Supplement Figure S2: Immunohistochemical expression in the two components of biphasic subtype and correlation with the percentage of epithelioid component.
