# Supplementary material for: New Immunohistochemical Markers for Pleural Mesothelioma Subtyping
Source: Diagnostics (Basel). 2023 Sep 14;13(18):2945. doi: 10.3390/diagnostics13182945 (PMC10529020; doi:10.3390/diagnostics13182945)
Supplement: Supplementary file 1 [file diagnostics-13-02945-s001.zip › Supplement Table S1.pdf]

Table S1. High immunohistochemical expression (> cut-off) among subtypes: Training and Validation cohort.

Training cohort					
	**Mesothelin**	**Claudin-15**	**CFB**	**PAI1**	**PAK4**
**Training cohort (n=73)**	37 (50.7%)	26 (35.6%)	25 (32.2%)	46 (63%)	54 (74%)
**ES (n=31)**	31 (100%)	21 (67.7%)	18 (58.1%)	11 (35.5%)	18 (58.1%)
**BS (n=25)**	6 (24%)	3 (12%)	7 (28%)	20 (80%)	23 (92%)
**SS (n=17)**	0 (0%)	2 (11.8%)	0 (0%)	15 (88.2%)	13 (76.5%)
Validation cohort					
	**Mesothelin**	**Claudin-15**	**CFB**	**PAI1**	**PAK4**
**Validation cohort (n=30)**	14 (46.7%)	11 (36.7%)	9 (30%)	20 (66.7%)	23 (76.7%)
**ES (n=11)**	10 (90.9%)	8 (72.7%)	7 (63.6%)	8 (72.7%)	7 (63.6%)
**BS (n=11)**	4 (36.4%)	3 (27.3%)	2 (18.2%)	6 (54.5%)	10 (90.9%)
**SS (n=8)**	0 (0%)	0 (0%)	0 (0%)	6 (75%)	6 (75%)

ES: epithelioid subtype; BS: biphasic subtype; SS: sarcomatoid subtype; CFB: Complement factor B; PAI1: Plasminogen activator inhibitor 1, PAK4: p21-activated kinase 4.
