# Supplementary figures and images for: New Immunohistochemical Markers for Pleural Mesothelioma Subtyping
Source: Diagnostics (Basel). 2023 Sep 14;13(18):2945. doi: 10.3390/diagnostics13182945 (PMC10529020; doi:10.3390/diagnostics13182945)

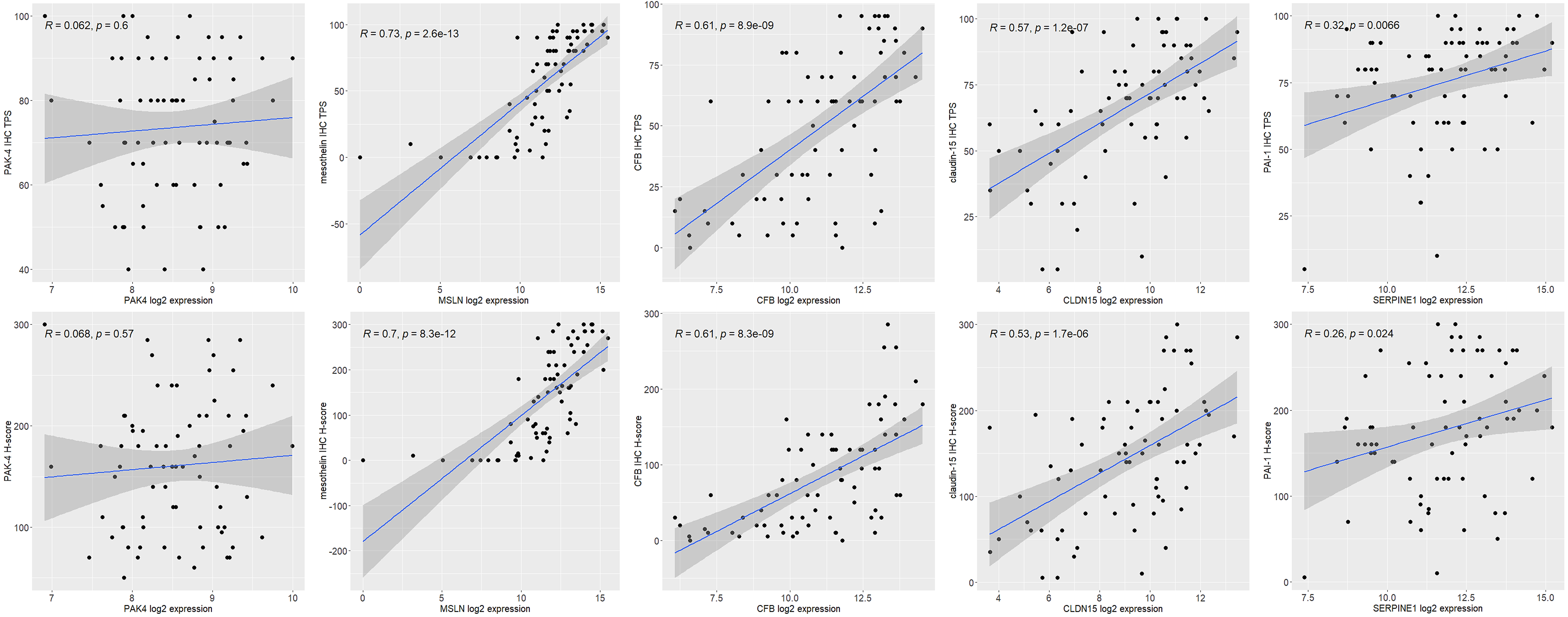

Supplement: Supplementary file 1 [file diagnostics-13-02945-s001.zip › Figure_S1.tif]

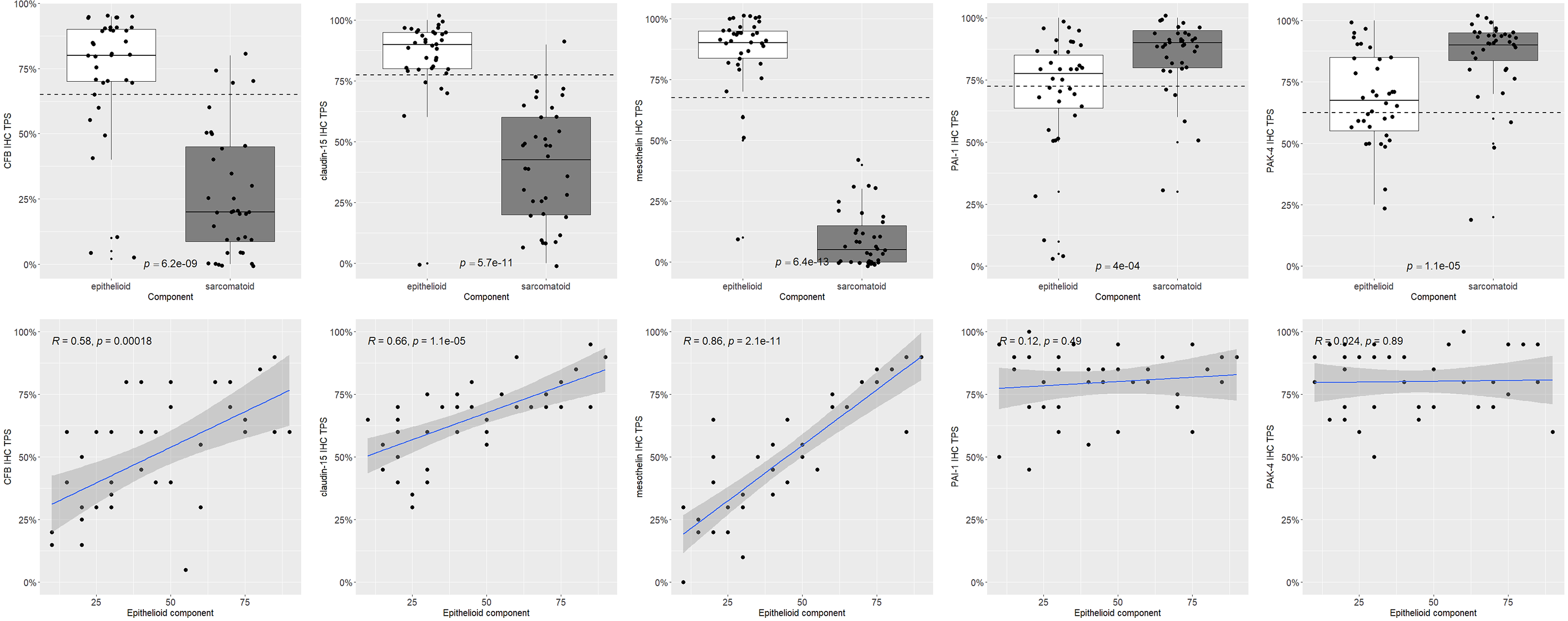

Supplement: Supplementary file 1 [file diagnostics-13-02945-s001.zip › Figure_S2.tif]
